# Metabolic evolution of *Corynebacterium glutamicum* for increased production of L-ornithine

**DOI:** 10.1186/1472-6750-13-47

**Published:** 2013-06-01

**Authors:** Ling-Yan Jiang, Shang-Guang Chen, Yuan-Yuan Zhang, Jian-Zhong Liu

**Affiliations:** 1 Biotechnology Research Centre and Biomedicine Centre, School of Life Sciences, Sun Yat-sen University, Guangzhou, 510275, People's Republic of China

**Keywords:** L-Ornithine, *Corynebacterium glutamicum*, Adaptive evolution, Metabolic engineering, Transcriptional level analysis

## Abstract

**Background:**

L-ornithine is effective in the treatment of liver diseases and helps strengthen the heart. The commercial applications mean that efficient biotechnological production of L-ornithine has become increasingly necessary. Adaptive evolution strategies have been proven a feasible and efficient technique to achieve improved cellular properties without requiring metabolic or regulatory details of the strain. The evolved strains can be further optimised by metabolic engineering. Thus, metabolic evolution strategy was used for engineering *Corynebacterium glutamicum* to enhance L-ornithine production.

**Results:**

A *C. glutamicum* strain was engineered by using a combination of gene deletions and adaptive evolution with 70 passages of growth-based selection. The metabolically evolved *C. glutamicum* strain, named ΔAPE6937R42, produced 24.1 g/L of L-ornithine in a 5-L bioreactor. The mechanism used by *C. glutamicum* ΔAPE6937R42 to produce L-ornithine was investigated by analysing transcriptional levels of select genes and NADPH contents. The upregulation of the transcription levels of genes involved in the upstream pathway of glutamate biosynthesis and the elevated NADPH concentration caused by the upregulation of the transcriptional level of the *ppnK* gene promoted L-ornithine production in *C. glutamicum* ΔAPE6937R42.

**Conclusions:**

The availability of NADPH plays an important role in L-ornithine production in *C. glutamicum.* Our results demonstrated that the combination of growth-coupled evolution with analysis of transcript abundances provides a strategy to engineer microbial strains for improving production of target compounds.

## Background

L-ornithine, a non-essential amino acid and an important constituent of the urea cycle, is the precursor of other amino acids, such as citrulline and arginine. It is effective for the treatment and prophylaxis of liver diseases [1], and has also been applied to wound healing [2]. Recently, it was demonstrated that L-ornithine supplementation increased serum levels of growth hormone and insulin-like growth factor-1 after heavy-resistance exercise in strength-trained athletes [3]. Many studies have reported that high yields of L-ornithine can be produced from a citrulline- or arginine-requiring mutant of a coryneform bacterium obtained by classical mutagenesis [4-7]. Although this mutant can produce a high yield of L-ornithine, its culture is unstable owing to reversion of the auxotrophic phenotype, which causes the production of L-ornithine to drop markedly.

Several recent reports have described progress in metabolic engineering of microorganisms for L-ornithine production. Lee and Cho reported that an engineered *Escherichia coli* produced 13.2 mg L-ornithine per gram of dry cell weight (DCW), and that addition of glutamate to the culture favoured L-ornithine production in the engineered *E. coli*[8]. Hwang et al. reported that co-overexpression of *argCJBD* in a triple-gene knockout strain *C. glutamicum* ATCC 13032 (*ΔargFΔargRΔproB*) resulted in a cellular L-ornithine content of 16.49 mg/g DCW and a concentration of L-ornithine in the culture medium of 179.14 mg/L [9]. Proline can be converted into L-ornithine by ornithine cyclodeaminase, which is a key enzyme responsible for enhancing L-ornithine production by *C. glutamicum* in proline-supplemented media [10]. Huang and Cho reported that over-expression of the Ncgl1469 open reading frame, exhibiting N-acetylglutamate synthase activity, increased L-ornithine production in *C. glutamicum* by 39% [11]. Recently, the same workers deleted the gluconate kinase gene *gntK* of *C. glutamicum* ATCC 13032 (*ΔargFΔargR*) to obtain *C. glutamicum* SJC8399, which produced 13.16 g/L of L-ornithine [12]. The L-ornithine producing strain *C. glutamicum* ATCC 13032 (*ΔargFΔargR*) named ORN1 was constructed and shown to produce L-ornithine from arabinose when *araBAD* from *E. coli* was expressed [13]. Recently, this group also constructed an engineered *C. glutamicum* ORN1 (pEKEx3-*xylA*_*Xc*_*-xylB*_*Cg*_) to effectively produce L-ornithine from xylose [14]. In our previous paper [15], we constructed a strain of *C. glutamicum* in which three genes had been deleted. This strain, named ATCC13032 (*ΔargFΔproBΔkgd*), produced L-ornithine of 18.17 g/L in the optimal medium [16].

Adaptive evolution strategies have been proven a feasible and efficient technique to achieve improved cellular properties without requiring metabolic or regulatory details of the strain [17-20]. The defining feature of adaptive evolution involves applying a selection pressure that favours the growth of mutants with the traits of interest. Growth-coupled adaptive evolution can significantly increase yields [21]. When combined with metabolic engineering, adaptive evolution is known as metabolic evolution engineering. The evolved strains can be further optimised by metabolic engineering. Metabolic evolution has been successfully employed for the improved production of succinate [20,22], L-alanine [23], and dihydroxyacetone [24]. However, to our knowledge, metabolic evolution engineering has never been reported to boost the production of L-ornithine.

In this study, we first deleted the *speE* gene of *C. glutamicum* ATCC 13032 (*ΔargFΔproB*) to obtain *C. glutamicum* ATCC 13032 (*ΔargFΔproBΔspeE*), which was then modified using growth-coupled adaptive evolution to improve L-ornithine production. After comparing the transcriptional levels of select genes of the evolved strain with those of the parent strain, additional genetic modifications were introduced to the evolved strain to further improve L-ornithine production. The mechanism of L-ornithine production by the metabolic evolved strain was also investigated by analysing transcriptional levels of genes of interest, as well as NADPH concentrations.

## Results

### Construction of the triple gene deletion ***C. glutamicum*** for adaptive evolution

In our previous paper [15], proteomic analysis demonstrated that the spermidine synthase encoded by the *speE* gene was more abundant in *C. glutamicum* engineered to overproduce L-ornithine than in wild-type *C. Glutamicum.* The upregulation might result in the degradation of L-ornithine. Thus, we deleted the *speE* gene of *C. glutamicum* (*ΔargFΔproB*) to obtain *C. glutamicum* ΔAPE. As expected, the deletion of the *speE* gene enhanced L-ornithine production. The *C. glutamicum* ΔAPE strain produced 11.3 ± 0.3 g/L of L-ornithine, which is higher than that of *C. glutamicum* (*ΔargFΔproB*) (10.2 ± 0.2 g/L; Table 1). Thus, *C. glutamicum* ΔAPE was used as the parent strain for adaptive evolution.

**Table 1:** L-Ornithine production by different strains

**Strain**	**OD**_**600**_	**L-Ornithine concentration (g/L)**	**L-Ornithine content (g/g DCW)**
*C*. *glutamicum* (*ΔargFΔproB*)	20.1 ± 0.7	10.2 ± 0.2	1.8 ± 0.1
*C*. *glutamicum*ΔAPE	18.5 ± 0.3^*^	11.3 ± 0.3^*^	2.2 ± 0.2^*^
*C*. *glutamicum* ΔAPE6937	23.5 ± 1.4^*^	13.6 ± 0.5^*^	2.1 ± 0.1
*C*. *glutamicum* ΔAPE6937(pEC-XK99E)	28.0 ± 1.2^#^	12.2 ± 0.3^#^	1.6 ± 0.1^#^
*C*. *glutamicum* ΔAPE6937(pEC-argB_cg_)	24.5 ± 1.0^#^	14.3 ± 0.5^#^	2.1 ± 0.2^#^
*C*. *glutamicum* ΔAPE6937(pEC-argB_ec_)	22.6 ± 1.9^#^	13.1 ± 0.2^#^	2.1 ± 0.2^#^
*C*. *glutamicum* ΔAPE6937R42	22.7 ± 0.1^*^	17.3 ± 0.4^*^	2.7 ± 0.2^*^

*Significantly different from the parent strain. ^#^Significantly different from ΔAPE6937(pEC-XK99E).

### Adaptive evolution

**Figure 1:**
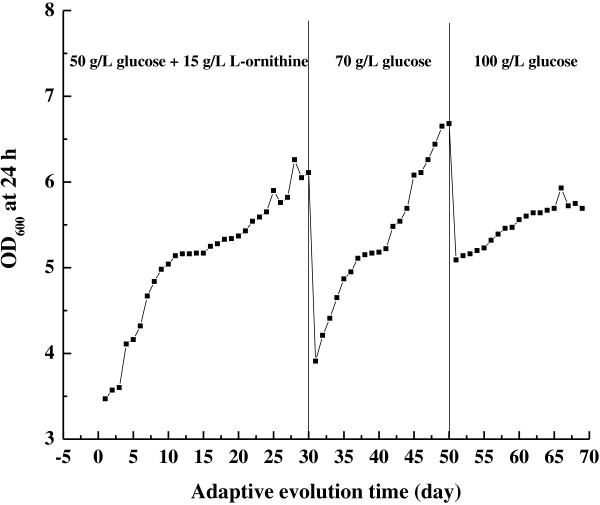
**Growth of *C*. *glutamicum *(*ΔargFΔproBΔspeE*) during adaptive evolution.**

To improve L-ornithine production, *C. glutamicum* ΔAPE was subjected to adaptive evolution driven by growth-based selection. This process comprised two stages (Figure 1). First, glucose and L-ornithine were added into the fermentation medium to overcome their inhibitions for growth and overproduction of metabolite. Screening a mutant resistant to substrate and end product is one common strategy for strain improvement by classical mutagenesis. At the later stage, only glucose was added at gradually increasing levels, and no L-ornithine was needed. After 70 days of adaptive evolution, one of the clones, referred to as *C. glutamicum* ΔAPE 6937, was chosen for further study. The *C. glutamicum* ΔAPE 6937 strain produced 13.6 ± 0.5 g/L of L-ornithine. The yield was about 20% higher than that of the parent strain *C. glutamicum* ΔAPE (11.3 ± 0.3 g/L; Table 1). However, the L-ornithine content of the evolved strain *C. glutamicum* ΔAPE 6937 was similar to that of the parent strain *C. glutamicum* ΔAPE. This suggests that the increased L-ornithine level of the evolved strain *C. glutamicum* ΔAPE 6937 is caused by the increased cell density.

### Characterisation of the evolved strain using qRT-PCR and sequence analysis

**Figure 2:**
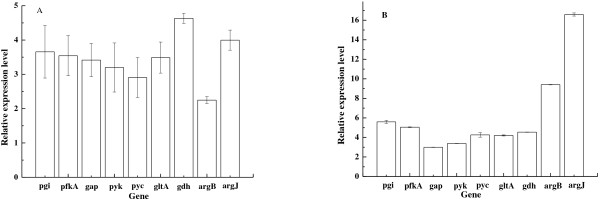
**Levels of select transcripts in the evolved strains *C*. *glutamicum *ΔAPE6937 (A) and *C*. *glutamicum *ΔAPE6937R42 (B) compared with those of the parent strain *C*. *glutamicum *ΔAPE at 54 h in shake flasks.** Abundances of each transcript in ΔAPE6937 or ΔAPE6937R42, determined using qRT-PCR, were normalised relative to levels of the same transcript in the parental strain.

In order to understand the mechanism of the increased L-ornithine level in the evolved strain, we analysed the transcriptional levels of genes that encode enzymes involved in L-ornithine biosynthesis in the evolved strain *C. glutamicum* ΔAPE6937. These genes comprise *pgi* (encoding glucose-6-phosphate isomerase), *pfkA* (encoding 6-phosphofructokinase), *gap* (encoding glyceraldehyde-3-phosphate dehydrogenase), *pyk* (encoding pyruvate kinase), *pyc* (encoding pyruvate carboxylase), *gltA* (encoding citrate synthase), *gdh* (encoding glutamate dehydrogenase), *argB* (encoding acetylglutamate kinase) and *argJ* (encoding the bifunctional ornithine acetyltransferase/N-acetylglutamate synthase). And then we compared the data with that obtained in the parent strain *C. glutamicum* ΔAPE. The results are presented in Figure 2A. All of these genes in the evolved strain *C. glutamicum* ΔAPE6937 are upregulated, with the smallest degree of upregulation seen for the *argB* gene.

In an attempt to identify mutations that confer the high-yield phenotype, we sequenced the above genes of the evolved strain ΔAPE6937 and compared the results with sequences of the same genes from *C. glutamicum* ATCC 13032. Analysis of the sequences of the *pgi, pfkA, gapA, pyk, pyc, gltA, gdh, argB*, and *argJ* genes in the evolved strain failed to identify any mutations relative to the parent strain.

### Genetic modification of the evolved strain

The results shown in Figure 2A suggest that expression of the *argB* gene may be the bottleneck for L-ornithine production by the evolved strain. Thus, we first over-expressed either of the *C. glutamicum* or *E. coli argB* genes in the evolved strain. Over-expression of either of the two *argB* genes indeed enhanced L-ornithine concentration and content, with over-expression of *C. glutamicum argB* increasing levels of L-ornithine concentration to a greater degree than that achieved by the over-expression of *E. coli argB* (Table 1).

The expression of the *arg* operon for control of the L-ornithine biosynthesis pathway is regulated by the arginine repressor ArgR. In addition, the DNA-binding affinity of ArgR to the upstream of *argB* gene was suggested to play an important role in L-ornithine biosynthesis in *C. glutamicum*[25]. Deletion of the *argR* gene is another strategy for enhancing the level of expression of the *arg* operon. Thus, we deleted the *argR* gene of the evolved strain *C. glutamicum* ΔAPE6937 to generate *C. glutamicum* ΔAPE6937R42. As expected, *C. glutamicum* ΔAPE6937R42 produced 17.3 ± 0.4 g/L of L-ornithine (Table 1). The concentration of L-ornithine was 27% higher than that of *C. glutamicum* ΔAPE6937.

To investigate the mechanism of L-ornithine production in *C. glutamicum* ΔAPE6937R42, we analysed the transcriptional levels of the genes that encode enzymes involved in L-ornithine biosynthesis in *C. glutamicum* ΔAPE6937R42 by using qRT-PCR, and compared the data with that obtained in *C. glutamicum* ΔAPE (Figure 2B). Deletion of the *argR* gene promoted the upregulations of the transcript levels of the *pgi, pfkA, argB*, and *argJ* genes. The respective transcriptional levels of the *pgi, pfkA, argB*, and *argJ* genes in *C. glutamicum* ΔAPE6937R42 are about 5.6-, 5.0-, 9.4-, and 16.6-fold (*p* < 0.05) higher than those in the parent strain *C. glutamicum* ΔAPE. In contrast, the respective levels of the same transcripts in *C. glutamicum* ΔAPE6937 are only about 3.7-, 3.5-, 2.3-, and 4.0-fold higher (*p* < 0.05) than those in the parent strain *C. glutamicum* ΔAPE (Figure 2A).

**Figure 3:**
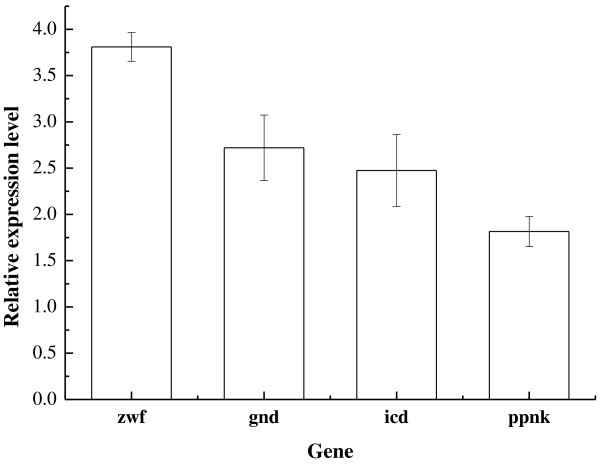
**Levels of transcripts involved in NADPH biosynthesis in *C*. *glutamicum *ΔAPE6937R42 compared with those in *C*. *glutamicum *ΔAPER grown at 54 h in shake flasks.** Abundances of transcripts in ΔAPE6937R42, determined using qRT-PCR, were normalised relative to levels of the same transcript in ΔAPER.

Three reactions in the L-ornithine biosynthesis pathway involve NADPH. These are the reactions catalysed by NADP-dependent isocitrate dehydrogenase, NADP-dependent glutamate dehydrogenase, and NADP-dependent N-acetyl-gamma-glutamyl-phosphate reductase. To analyse the effect of NADPH availability on L-ornithine accumulation, we deleted the *argR* gene of the parent strain *C. glutamicum* ΔAPE to obtain *C. glutamicum* ΔAPER, and then analysed the NADPH contents of the two strains. As expected, *C. glutamicum* ΔAPE6937R42 produced more NADPH and L-ornithine than *C. glutamicum* ΔAPER (Table 2). To better understand the effect of NADPH, we compared the transcript levels of the genes involved in NADPH synthesis in the *C. glutamicum* strains ΔAPE6937R42 and ΔAPER. The results are presented in Figure 3. In *C. glutamicum* ΔAPE6937R42, the genes involved in NADPH synthesis (*zwf, gnd*, and *icd*) were upregulated by 3.8-, 2.7- and 2.5-fold, respectively (*p* < 0.05). The *ppnk* gene was also upregulated by 1.8-fold (*p* < 0.05). Therefore, we examined whether the increased NADPH levels are caused by the upregulations of the transcriptional levels of these genes in *C. glutamicum* ΔAPE6937R42. We over-expressed these genes in *C. glutamicum* ΔAPER. The results are presented in Table 3. Over-expression of the *zwf*, *gnd* and *ppnK* genes indeed increased the concentration of NADPH in *C*. *glutamicum* ΔAPER. However, only over-expression of the *ppnK* gene promoted L-ornithine production. It suggests that only the increased NADPH level caused by the elevated transcriptional level of the *ppnK* gene promoted L-ornithine production.

**Table 2:** Concentrations of NADPH in the different strains

**Strain**	**OD**_**600**_	**NADPH (μM)**	**L-ornithine concentration (g/L)**
*C*. *glutamicum* ΔAPE6937R42	22.4 ± 1.0	34.2 ± 0.2	17.0 ±0.6
*C*. *glutamicum* ΔAPER	13.7 ± 0.3	11.6 ± 0.2	12.4 ± 0.6

**Table 3:** **Effect of over-expression of gene in *C*. *glutamicum *ΔAPER on L-ornithine production**

**Plasmid**	**OD**_**600**_	**NADPH (μM)**	**L-Ornithine concentration (g/L)**
pEC-XK99E	14.6 ± 0.1^*^	1.2 ± 0.15^*^	14.2 ± 0.4^*^
pEC-zwf	14.3 ± 1.3	6.1 ± 0.2^*^	14.6 ± 0.5
pEC-ppnk	11.1 ± 0.6^*^	6.2 ± 0.2^*^	15.4 ± 0.4^*^
pEC-gnd	14.6 ± 1.6	3.1 ± 0.2^*^	14.4 ± 0.5
pEC-pntAB	22.9 ± 1.0^*^	7.4 ± 0.4^*^	17.6 ± 0.4^*^

*Significantly different from the control strain.

### Fermentation of the strain generated by metabolic evolution

**Figure 4:**
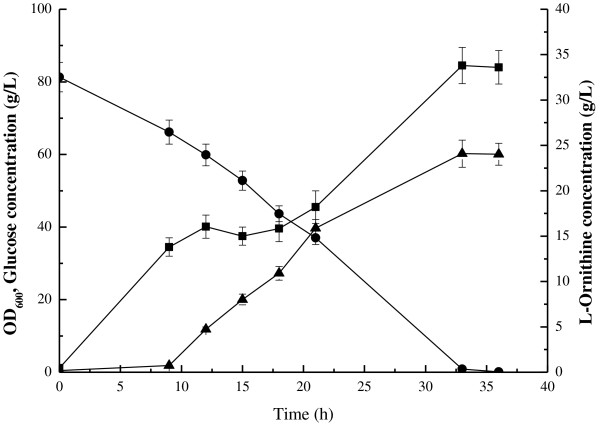
**Batch culture of *C*. *glutamicum *ΔAPE6937R42 in 5 L bioreactor.** (●) glucose; (■) OD_600_; (▲) L-ornithine.

For a more detailed view on L-ornithine production, *C*. *glutamicum* ΔAPE6937R42 was cultured in a 5-L bioreactor (Figure 4), and was found to grow in a diauxic manner. The maximum L-ornithine concentration (24.1 ± 1.5 g/L) and yield (0.3 g/g) was obtained at 33 h, at which time all glucose in the medium had been consumed.

## Discussion

In this study, we first deleted the *speE* gene to enhance L-ornithine production (Table 1). Spermidine synthase encoded by *speE* catalyzes the formation of spermidine from putrescine. Although the genes involved in the biosynthesis of putrescine remain unknown, cells of *C*. *glutamicum* contain putrescine and polyaminies [26]. The deletion of the *speE* gene blocked putrescine to be converted into spermidine and might alleviate the degradation of L-ornithine. However, the real reason of promoting L-ornithine production by the deletion of the *speE* gene should be further investigated.

Sequence data of the nine genes of *C*. *glutamicum* ΔAPE6937 we characterised at the transcript level failed to uncover any mutations in the evolved strain. It is possible that mutations in other genes may have conferred the observed phenotypes. These might include other genes that encode enzymes responsible for L-ornithine biosynthesis or transcription factors that regulate the expression of the sequenced genes. Kutyna et al. also reported that there were no apparent mutations in the *Saccharomyces cerevisiae* B2-c3 they evolved to generate elevated yields of glycerol [27]. Further work will be required to identify the genetic determinants of these traits.

In *C*. *glutamicum* ΔAPE6937 and ΔAPE6937R42, elevated transcriptional levels of the genes involved in the upstream pathway of glutamate biosynthesis (*pgi*, *pfkA*, *gap*, *pyk*, *pyc*, *gltA* and *gdh*, Figure 2) indicated increased availability of endogenous glutamate compared with the parental strain. Pyruvate kinase is a major bottleneck for glutamate production, and over-expression of the *pyc* gene improved glutamate production in *C*. *glutamicum* ATCC 13032 [28]. Shirai et al. reported that the fluxes of the reactions catalysed by Pgi, PfkA, Gap, Pyk, Pyc, GltA, and Gdh were increased with glutamate production in *C*. *glutamicum*[29]. In our previous paper [15], these enzymes were more abundant in *C*. *glutamicum* engineered to overproduce L-ornithine than in wild-type *C*. *glutamicum*. It is thought that increasing the availability of endogenous glutamate might increase L-ornithine production. Lee and Cho reported that the availability of glutamate was a rate-limiting factor in L-ornithine accumulation, and that addition of glutamate in the media increased L-ornithine production in engineered *E*. *coli*[8]. However, the same group reported that the intracellular concentration and supply of glutamate is not a rate-limiting step for L-ornithine production in an L-ornithine-producer *C*. *glutamicum* SJ8074 owing to the presence of rate limiting steps in L-ornithine biosynthesis downstream of glutamate synthesis in *C*. *glutamicum* SJ8074 [9]. They also reported that the increased availability of glutamate might increase L-ornithine accumulation in certain genetic backgrounds (e.g., that of the L-ornithine-producer *C*. *glutamicum* SJ8039) [9]. The transcriptional levels of the *argB* and *argJ* genes were much higher than those of the genes that encode enzymes that act upstream of glutamate in *C*. *glutamicum* ΔAPE6937R42 (Figure 2B). This indicates that there is no rate-limiting step in L-ornithine synthesis downstream of the synthesis of glutamate in *C*. *glutamicum* ΔAPE6937R42. Thus, the elevated transcriptional levels of the genes involved in the upstream pathway of glutamate biosynthesis may be one reason of the increased L-ornithine production in *C*. *glutamicum* ΔAPE6937R42.

This study demonstrated that NADPH availability and L-ornithine production are strongly correlated. Production of L-ornithine requires 2 mol of NADPH per mole of L-ornithine produced by *C*. *glutamicum*. Increases in the levels of *zwf*, *gnd*, and *icd* transcripts can increase NADPH availability in *C*. *glutamicum* ΔAPE6937R42. However, increased levels of *zwf* and *gnd* transcripts can drive the carbon metabolic flux from the Embden–Meyerhof–Parnas pathway to the pentose phosphate (PP) pathway. Moreover, given that the PP pathway is coupled with CO_2_ production, direct enhancement of the PP pathway may result in release of CO_2_, thereby decreasing the yield of L-ornithine. Thus, enhanced carbon flux through the PP pathway, caused by the upregulation of *zwf* and *gnd* at the transcriptional level, inhibits L-ornithine production. Our results also demonstrated this point. Over-expression of the *zwf* and *gnd* genes could not increase L-ornithine production in *C*. *glutamicum* ΔAPER although it could enhance NADPH concentration (Table 3). These suggest that the increased level of L-ornithine production in the evolved strain *C*. *glutamicum* ΔAPE6937R42 was not caused by the increased levels of *zwf* and *gnd* transcripts.

ATP-dependent NAD kinase encoded by *ppnK* catalyzes the phosphorylation of NAD to NADP. Increased transcriptional levels of *ppnK* increased the size of the NADP pool, thus potentially increasing the abundance of NADPH. Our results demonstrated that the increased level of *ppnK* transcript indeed enhanced the abundance of NADPH in the evolved strain *C*. *glutamicum* ΔAPE6937R42 (Figure 3 and Table 2). Lindner et al. reported that over-expression of *ppnk* improved L-lysine production in *C*. *glutamicum* by 12% [30]. Overexpression of *nadk*, which encodes NAD kinase, increased the NADPH/NADP ratio, which in turn enhanced thymidine biosynthesis in *E*. *coli*[31]. Our result also demonstrated that over-expression of the *ppnK* gene enhanced L-ornithine production in *C*. *glutamicum* ΔAPER. Thus, the elevated level of *ppnK* transcript that increase the availability of NADPH may be another reason of increased L-ornithine production in *C*. *glutamicum* ΔAPE6937R42.

Other strategies have been developed to improve NADPH availability. Over-expression of *E*. *coli pntAB* genes, which encode a membrane-bound transhydrogenase enhanced NADPH availability, and thus increased L-lysine levels in *C*. *glutamicum*[32]. In this study, over-expression of *E*. *coli pntAB* genes enhanced NADPH availability (about 5.2-fold), and thus increased L-ornithine levels (23.7%) in *C*. *glutamicum*ΔAPER (Table 3). Simultaneous chromosomal overexpression of transhydrogenase (*pntAB*) and NAD kinase (*yfjB*) genes had a effect on increasing NADPH supply and improving anaerobic isobutanol production [33]. Replacement of the endogenous NAD-dependent glyceraldehyde 3-phosphate dehydrogenase with the NADP-dependent glyceraldehyde 3-phosphate dehydrogenase from *Streptococcus mutans* also increased both NADPH availability and L-lysine production in *C*. *glutamicum*[34]. The inactivation of the gluconate kinase gene (*gntK*) led to a 51.8% increase in intracellular NADPH concentration and a 49.9% increase in L-ornithine production [12]. These strategies may be useful for further improving L-ornithine production in *C*. *glutamicum* ΔAPE6937R42.

To the best of our knowledge, this is the first report of the use of metabolic evolution engineering to increase production of L-ornithine by *C*. *glutamicum*. The yield of our engineered stain (24.1 g/L) is unprecedented in any engineered *C*. *glutamicum* strain of which we are aware, including that described in our previous paper (18.17 g/L) [16] and that reported by Hwang and Cho (13.16 g/L) [12]. To date, the highest titre of L-ornithine reported in the literature was 74 g/L, as reported by Lee et al. [6]. Those researchers achieved this yield by using a 7-L fed-batch fermentation process with a glucose-feeding strategy and an L-arginine auxotrophic mutant of *Brevibacterium ketoglutamicum* ATCC 21092. The titre of this auxotrophic mutant, which was generated using classical mutagenesis, was only 2 g/L in batch culture [35]. This suggests that the yield of the new strains described in this study may be enhanced by growth using fed-batch fermentation technology.

## Conclusion

We first deleted the *speE* gene of *C*. *glutamicum* ATCC 13032 (*ΔargFΔproB*), then evolved by a growth-based selection process for 70 passages to generate *C*. *glutamicum* ΔAPE6937, and finally deleted the *argR* gene of the evolved strain to obtain *C*. *glutamicum* ΔAPE6937R42. The *C*. *glutamicum* ΔAPE6937R42 strain produced 24.1 g/L of L-ornithine in a 5-L bioreactor, a level unprecedented in any engineered strain. It has been demonstrated that the increased L-ornithine production in *C*. *glutamicum* ΔAPE6937R42 is dependent on the increased availabilities of glutamate caused by the elevated levels of transcripts involved in the upstream pathway of glutamate biosynthesis and the increased availabilities of NADPH caused by the elevated level of *ppnK* transcript. The availability of NADPH plays an important role in L-ornithine production in *C*. *glutamicum*.

## Methods

### Strains, primers and plasmids

All strains and plasmids used in this study are listed in Table 4. The *C*. *glutamicum* strain ATCC 13032 (*ΔargFΔproB*) [15] was used as the starting strain for L-ornithine production. Primers used in this study are listed in Table 5. L-ornithine was purchased from Sigma.

**Table 4:** Strains and plasmids used in this study

**Strain, plasmid**	**Properties/sequence**	**Source/reference**
Strain		
*Escherichia coli* DH5α	supE44, hsdR17, recA1, thi-1, endA1, lacZ, gyrA96, relA1	Invitrogen
*C*. *glutamicum*		
ATCC 13032	Wild-type	ATCC
ATCC 13032 (*ΔargFΔproB*)	*C*. *glutamicum* ATCC 13032, *ΔargF*, Δ*proB*	15
*Δ*APE	*C*. *glutamicum* ATCC 13032, *ΔargF*, Δ*proB*, *ΔspeE*	This study
*Δ*APE6937	The evolved strain of ATCC 13032 (*ΔargFΔproBΔspeE*)	This study
*Δ*APE6937R42	*Δ*APE6937, *ΔargR*	This study
*Δ*APER	*C*. *glutamicum* ATCC 13032, *ΔargF*, Δ*proB*, *ΔspeE*, *ΔargR*	This study
pK18mobsacB	*sacB*, *lacZa*, Km^r^, mcs mobilizable vector, allows for selection of double crossover *C*. *glutamicum*	39
pK-JL	pK18mobsacB derivative, *sacB* under the control of *tac-M* promoter, Km^r^,	This study
pMD18-T	TA cloning vector, Amp^r^	TaKaRa
pK-ΔargR	pK-JL with 506 bp deletion of the *argR* gene	This study
pEC-XK99E	*C*. *glutamicum*-*E*. *coli* shuttle expression vector, Kan^r^	41
pEC-argB_CG_	pEC-XK99E containing the *argB* gene from *C*. *glutamicum*	This study
pEC-argB_EC_	pEC-XK99E containing the *argB* gene from *E*. *coli*	This study
pEC-zwf	pEC-XK99E containing the *zwf* gene from *C*. *glutamicum*	This study
pEC-ppnK	pEC-XK99E containing the *ppnk* gene from *C*. *glutamicum*	This study
pEC-gnd	pEC-XK99E containing the *gnd* gene from *C*. *glutamicum*	This study
pEC-pntAB	pEC-XK99E containing the *gnd* gene from *E*. *coli*	This study

**Table 5:** Primers used in this study

**Primer**	Sequence*	**Purpose**
sacBF	cggcgactagttgagctgttgacaattaatcatcgtgtggtaccatgtgtggaattgtgagcggataacaattccgcgggttctttaggcccgtagtct (*Spe*I,*Sac*II)	PCR for the *sacB* gene
sacBR	gccgcgatatctctcgtgatggcaggtt (*Eco*RV)	PCR for the *sacB* gene
pk18msF	gcgccgatatcgttcgtctggaaggcagta (*Eco*RV)	PCR for the backbone of pK18mobsacB except for the *sacB* gene
pk18msR	gcgcgactagtgcatgggcataaagttgc (*Spe*I)	PCR for the backbone of pK18mobsacB except for the *sacB* gene
argR-F5	cgct*ggatcc*tttaagcacggcgttattt (*Bam*HI)	PCR for the upstream fragment of the *argR* gene
argR-R5	cgg*tctaga*tgcgagtcacgggattta (*Xba*I)	PCR for the upstream fragment of the *argR* gene
argR-F3	cgg*tctaga*ggtaaggtataacccgagtgt (*Xba*I)	PCR for the downstream fragment of the *argR* gene
argR-R3	cgat*gtcgac*gacttgatgcccacgaga (*Sal*I)	PCR for the downstream fragment of the *argR* gene
GargBF	cgctctagaaaggacacaggcatgaatgact (*Xba*I)	PCR for the *argB* gene from *C*. *glutamicum*
GargBR	cgggtcgacttacagttccccatcctt (*Sal*I)	PCR for the *argB* gene from *C*. *glutamicum*
EargBF	cgttctagaaggaggggtgcaatgatgaat (*Xba*I)	PCR for the *argB* gene from *E*. *coli*
EargBR	gcggtcgaccttaagctaaaatccg (*Sal*I)	PCR for the *argB* gene from *E*. *coli*
zwf-F	ccgcctctagaaaggagaccatcatgagcacaaacac (*Xba*I)	PCR for the *zwf* gene from *C*. *glutamicum*
zwf-R	cggtagtcgacccctaaattatggcctgc (*Sal*I)	PCR for the *zwf* gene from *C*. *glutamicum*
ppnK-F	gccatgaattcaaggacgcaataatgactgcacccacgaa (*EcoR*I)	PCR for the *ppnK* gene from *C*. *glutamicum*
ppnK-R	ccgccgagctccgaattaccccgctgac (*Sac*I)	PCR for the *ppnK* gene from *C*. *glutamicum*
gnd-F	gcgatggtaccaaggagaccactatgccgtcaagtacgat(*Kpn*I)	PCR for the *gnd* gene from *C*. *glutamicum*
gnd-R	ccgcgtctagaaaaggagagcctttaagct (*Xba*I)	PCR for the *gnd* gene from *C*. *glutamicum*
pntAB-F	cagggtacctcatcaataaaaccg(*Kpn*I)	PCR for the *pntAB* gene from *E*. *coli*
pntAB-R	cgtctgcagttacagagctttcag(*Pst*I)	PCR for the *pntAB* gene from *E*. *coli*
qpgiF	cccttctattctcggtgc	qRT-PCR for *pgi*
qpgiR	aggtcatttgcctgctgt	qRT-PCR for *pgi*
qpfkAF	tatccctgttgtcggtgtc	qRT-PCR for *pfkA*
qpfkAR	gtgagattcagcggtggt	qRT-PCR for *pfkA*
qgapF	ggaagttgaatacgacgatga	qRT-PCR for *gap*
qgapR	gcccagtccaggttcttt	qRT-PCR for *gap*
qpycF	accgccacgaaatccc	qRT-PCR for *pyc*
qpycR	aacggctgcgtagttgtct	qRT-PCR for *pyc*
qpykF	ccgtgcagtcggtattct	qRT-PCR for *pyk*
qpykR	gcgttccctctacatcgt	qRT-PCR for *pyk*
qgltAF	cgggaatcctgcgttac	qRT-PCR for *gltA*
qgltAR	tggcgaatctcgtcgtt	qRT-PCR for *gltA*
qgdhF	ccgccacatcggtgagta	qRT-PCR for *gdh*
qgdhR	agccatgcgacggtagt	qRT-PCR for *gdh*
qargBF	ggtttggtcggagacatca	qRT-PCR for *argB*
qargBR	gcctggagcaatcgtagag	qRT-PCR for *argB*
qargJF	cctgacatggcgttgg	qRT-PCR for *argJ*
qargJR	ctcggctcaccttcaca	qRT-PCR for *argJ*
qzwfF	acccgcaggataaacga	qRT-PCR for *zwf*
qzwfR	gctagatcataaatggc	qRT-PCR for *zwf*
qppnkF	gtttaccgaccgacttgtg	qRT-PCR for *ppnk*
qppnkR	gctgacctgggatctttatt	qRT-PCR for *ppnk*
qicdF	aggaccagggctacgacat	qRT-PCR for *icd*
qicdR	gcggaacccttaacagc	qRT-PCR for *icd*
qgndF	aaccgcagcactgacaaa	qRT-PCR for *gnd*
qgndR	cagggatgctacgaactct	qRT-PCR for *gnd*
16s-F	tcgatgcaacgcgaagaac	qRT-PCR for *16srRNA*
16s-R	gaaccgaccacaagggaaaac	qRT-PCR for *16srRNA*

*Restriction enzyme sites are underlined.

### L-ornithine production in shake flasks

For L-ornithine fermentations, a 1.0-mL sample of the seed culture that had been grown at 150 rpm and 30°C for 12 hours was inoculated into 10 mL of the fermentation medium in a 100-mL flask and incubated at 30°C and 150 rpm for 72 hours. Each litre of the seed medium contained 25 g of glucose, 10 g of yeast extract, 10 g of corn steep liquor, 15 g of (NH_4_)_2_SO_4_, 2.5 g of MgSO_4_·7H_2_O, 1 g of KH_2_PO_4_, 0.5 g of K_2_HPO_4_, 0.5 g of Na_2_HPO_4_, and 10 g of CaCO_3_. Each litre of the fermentation medium contained 100 g of glucose, 20 g of corn steep liquor, 50 g of (NH_4_)_2_SO_4_, 2.5 g of MgSO_4_·7H_2_O, 1 g of KH_2_PO_4_, 0.5 g of K_2_HPO_4_, 0.5 g of Na_2_HPO_4_, 20 mg of FeSO_4_·7H_2_O, 20 mg of MnSO_4_·4H_2_O, 2 g of molasses, 1 mL of Tween-80, and 10 g of CaCO_3_. The initial pH of both media described above was adjusted to 7.0.

### Adaptive evolution

The adaptive evolution process is presented in Figure 1. Adaptive evolution was conducted in a test tube that contained 5 mL of fermentation medium supplemented with 50 g/L glucose and 15 g/L L-ornithine, and incubated at 30°C and 200 rpm. Cultures were serially passed into fresh medium (initial OD_600_ of 0.2) daily. After repeating this transfer procedure 30 times, the culture was then sequentially transferred to fermentation medium containing 70 g/L glucose. The daily transfer procedure at the glucose concentration was repeated 20 times. Finally, the culture was then sequentially transferred to fermentation medium with 100 g/L glucose, and the daily transfer procedure was repeated 20 times. Cultures were frozen and stored at −80°C at every 10 passages throughout adaptive evolution.

After the 70-day adaptive evolution process, cultures stored at −80°C were spread onto LB medium plates (10 g/L tryptone, 5 g/L yeast extract, 10 g/L NaCl). Single colony was transferred to MM medium plates (5 g/L of glucose, 1 g/L of (NH_4_)_2_SO_4_, 0.5 g/L of sodium citrate, 0.1 g/L of MgSO_4_·7H_2_O, 4.5 g/L K_2_HPO_4_, and 10.5 g of KH_2_PO_4_ (pH 7.0)) that was supplemented with 5 g/L L-ornithine and 5 g/L L-arginine. Only colonies that grew on the LB medium plates were further cultured in shake flasks to evaluate their levels of L-ornithine production.

### Quantitative real-time PCR (qRT-PCR)

Total RNA from *C*. *glutamicum* cells grown for 54 h in shake flasks was isolated using an RNA extraction kit (Dongsheng Biotech, Guangzhou, China), following the manufacturer’s instructions. The first-strand cDNA was synthesized using an All-in-One™ First-Strand cDNA Synthesis Kit (GeneCopoeia, Guangzhou, China). The qRT-PCR was performed with the All-in-One™ qPCR Mix kit (GeneCopoeia, Guangzhou, China) on an iCycler iQ5 Real Time PCR system (Bio-Rad Laboratories, USA). 100 ng of cDNA was used as template. The PCR conditions were: 95°C for 10 min, followed by 45 cycles of denaturation at 95°C for 10 s, annealing at 60°C for 20 s, and extension at 72°C for 15s. The primers for qRT-PCR are presented in Table 5. The quantification technique used to analyse data was the 2^-ΔΔCt^ method described by Livak and Schmittgen [36]. The data were normalized using expression of 16S rRNA.

### Gene knockout

Chromosomal DNA of *C*. *glutamicum* was isolated as described by Eikmanns et al. [37]. The preparation of competent cells and electroporation for *C*. *glutamicum* was performed as described by Van de Rest et al. [38]. The correct mutants of *C*. *glutamicum* were confirmed by PCR.

The lethality of the *sacB* gene in corynebacteria depends on its expression levels. Therefore, a 1.85-kb DNA fragment containing the *sacB* gene cluster was amplified by PCR using the primers sacBF and sacBR (Table 5), and the plasmid pK18mobsacB [39] as template. This converted the native promoter of the *sacB* gene cluster to a *tac*-*M* promoter, which is a strong promoter in corynebacteria [40]. The entire backbone of pK18mobsacB, except for the *sacB* gene, was amplified using the primers pk18msF and pk18msR (Table 5). The two fragments were digested with *Spe*I and *Eco*RV, and ligated together to form the inducible suicide vector pK-JL (5,570 bp).

Disruption of the gene was performed using the non-replicable integration vector pK-JL, which allows for marker-free deletion of the target gene [39]. The flanking sequence of the *argR* gene (1,153 bp and 921 bp) was amplified from the genomic DNA of *C*. *glutamicum* using the primers argR-F5/argR-R5 and argR-F3/argR-R3, and ligated separately into pMD18-T. The two fragments were excised using *Bam*HI/*Xba*I and *Xba*I/*Sal*I, respectively, and then ligated into the *Bam*HI/SalI sites of pK-JL to obtain the non-replicable integration vector pK-ΔargR, which contains an internal 506-bp deletion in the *argR* gene. The above nonreplicable integration vector pK-ΔargR was transferred into *C*. *glutamicum* to disrupt the site-specific gene using the protocol described by Schäfer et al. [39].

### Plasmid construction

The *argB* gene, which encodes acetylglutamate kinase, was amplified from the genomic DNA of *C*. *glutamicum* and *E*. *coli*, using the primer pairs GargBF/GargBR and EargBF/EargBR, respectively. The fragments were ligated separately into the pMD18-T vector. Both fragments were excised using *Xba*I/*Sal*I and then inserted into the *Xba*I/*Sal*I sites of pEC-XK99E [41] to obtain the over-expression vectors pEC-argB_CG_ and pEC-argB_EC_. The *zwf*, *ppnK* and *gnd* gene was amplified from the genomic DNA of *C*. *glutamicum* using the corresponding primer pairs (Table 5), and then inserted into the corresponding sites of pEC-XK99E, respectively. The *pntAB* genes were amplified from the genomic DNA of *E*. *coli*, using the primer pair pntAB-F/pntAB-R (Table 5) and then inserted into the *Kpn*I/*Pst*I sites of pEC-XK99E.

### NADPH assay

After aerobic cultivation of *C*. *glutamicum* on a rotary shaker (150 rpm) at 30°C for 54 h, the cells were harvested by centrifugation and washed twice with water. Intracellular NADPH was extracted and quantified using the Enzychrom^TM^ NADP^+^/NADPH Assay kit (BioAssay Systems, Hayward, CA) following the manufacturer’s instructions.

### Batch culture in bioreactor

Batch culture was carried out at 30°C in a 5-L jar fermentor (Biostat B5, B. Braun, Germany) that contained 3 L of fermentation medium. To prepare the inocula, 10 mL of LB medium was inoculated with a small aliquot of cell glycerol stock that had been stored at −80°C, and was cultured overnight at 30°C. One millilitre of the overnight culture was subsequently transferred into a 250-mL Erlenmeyer flask containing 50 mL of the seed medium, and incubated for 24 h at 30°C and 150 rpm in a shaking incubator. The seed cultures (300 mL) were inoculated into the fermenter for batch cultivation. The pH was maintained at 7.0 by adding NH_4_OH. Antifoam was added manually as needed. The aeration rate was 1.0 L/L/min and the agitation rate was 400 rpm. Samples were periodically taken for the measurements of OD_600_, residual glucose concentration, and L-ornithine concentration. Fermentation experiments were carried out in duplicate.

### Assays of cell growth, L-ornithine, and glucose

Cell growth was monitored by measuring the optical density of the culture at 600 nm (OD600) using a spectrophotometer (Shimadzu Corporation, Japan) after dilution of the culture with 0.2 mol/L HCl to dissolve CaCO_3_. L-Ornithine concentrations were determined by colorimetry, using ninhydrin as described previously [42]. Glucose concentration was determined by glucose oxidase using a glucose assay kit (Shanghai Rongsheng Biotech Corporation, China).

### Statistical analysis

All experiments were conducted in triplicate, and data were averaged and presented as the mean ± standard deviation (SD). One-way analysis of variance (ANOVA) followed by Tukey’s test was used to determine significant differences using OriginPro (version 7.5) software. Statistical significance was defined as *p* < 0.05.

## Competing interests

The authors declare that they have no competing interests.

## Authors’ contributions

LY J carried out most of the experiments. SG C carried out fermentation in 5L bioreactor. YY Z constructed some expression vectors. JZ L developed the concept and designed the method, led the project and drafted the manuscript. All authors read and approved the final manuscript.
